# Epidemiology of clinical congenital and neonatal malaria in endemic settings: a systematic review and meta-analysis

**DOI:** 10.1186/s12936-020-03373-8

**Published:** 2020-08-28

**Authors:** Celestin Danwang, Jean Joel Bigna, Rolf Nyah Tuku Nzalie, Annie Robert

**Affiliations:** 1 Epidemiology and Biostatistics Unit, Institut de Recherche Expérimentale et Clinique, Université catholique de Louvain, Brussels, Belgium; 2 Department of Epidemiology and Public Health, Centre Pasteur of Cameroon, Yaoundé, Cameroon; 3 School of Public Health, University of Buea, Buea, Cameroon

**Keywords:** Epidemiology, Malaria, Neonates, Review

## Abstract

**Background:**

In order to attain the objectives set out in the global technical strategy against malaria 2016–2030, it is important to have accurate epidemiological data on malaria in all age categories, including those which are often neglected because of an apparent low burden of disease. The current systematic review with meta-analysis synthesizes the epidemiology of clinical congenital and neonatal malaria in endemic areas.

**Methods:**

PubMed, EMBASE, Global Index Medicus, and Web of Science were searched up to 30th October 2019, to identify observational studies reporting on congenital (0–7 days) and neonatal (0–28 days) malaria. No restriction related to language was applied. Study selection, data extraction, and methodological quality assessment were performed independently by two investigators. A random-effects meta-analysis was used to pool prevalence data. Prevalence were adjusted taking into account the variance due to diagnostic method and regional distribution. Subgroup analyses were performed to identify sources of heterogeneity in case of substantial heterogeneity. This review was registered in PROSPERO with number CRD42020150124.

**Results:**

The bibliographical search identified 1,961 studies, of which 22 were finally retained with a total population of 28,083 neonates. The overall crude prevalence of clinical congenital malaria was 40.4‰ (95%CI 19.6**–**67.7; 17 studies). The adjusted prevalence considering the variance due to difference in region/country (hierarchical model) was 33.7‰ (95%CI 6.9**–**77.2). There was no difference between the prevalence of clinical congenital malaria in Africa 39.5‰ (95%CI 17.2**–**59.5; 15 studies) and outside Africa 56.3‰ (95%CI 0.0**–**406.1), *p* = 0.867. The overall crude prevalence of clinical neonatal malaria was 12.0‰ (95%CI 1.4**–**30.3; 12 studies), and the adjusted one (considering the variance due to diagnostic method and the region/country) was 12.9‰ (95%CI 0.1**–**39.7). There was no difference between the prevalence of clinical neonatal malaria in Africa 12.1‰ (95%CI 1.3**–**31.2; 11 studies) and outside Africa 12.5‰ (95%CI 0.0**–**52.9), *p* = 0.802.

**Conclusion:**

This study suggests a high prevalence of clinical congenital and neonatal malaria. It calls for an intensification of preventive measures against malaria during pregnancy and in the neonatal period, and to consider neonates as a distinct age category in the elaboration of malaria treatment and prevention guidelines.

## Background

During the past decade, considerable efforts have been made to reduce the global burden of malaria [1–3]. These efforts have led to significant reductions in malaria-related morbidity and mortality [1–5]. Indeed, the World Health Organization (WHO) has estimated that the incidence of malaria has been reduced by 22% over the last 8 years in sub-Saharan Africa, while the figure is close to 70% in South-East Asia [1].

Although in some continents, several countries have eradicated malaria as endemic infectious disease in the last 50 years, other countries still remain endemic to malaria with varying trends over time, especially in sub-Saharan Africa [6–11]. Indeed, in 2018, 19 sub-Saharan African countries and India bore 85% of the world’s malaria burden, with 94% of malaria-related death occurring in sub-Saharan Africa [1]. These data point out difficulties in achieving the global technical strategy against malaria 2016–2030, which aims to reduce malaria morbidity by at least 40% in 2020 [1] This calls for intensified efforts, particularly in the regions of the world most affected by malaria.

Assessment of the level of achievement of malaria control and eradication goals has been possible in recent years thanks to the presence of high-quality global data. Enabling the assessment of the global burden of malaria often at very high spatial resolutions [2, 4, 5]. Nevertheless, among all published studies reporting on malaria, many focus on adults, pregnant women and children under 5 years of age [4, 5, 12]. Yet this last age category includes several heterogeneous groups with different immunological characteristics and varying susceptibility to infection [13–15].

While it is recognized that pregnant women and children under 5 years of age are the two categories of the population most vulnerable to malaria, data for children under 5 years of age need to be disaggregated because they include subgroups (neonates, infants, toddlers and early childhood children) that have different immunological characteristics and host-parasite interactions [13–18].

Currently, in countries with high malaria endemicity, the diagnosis of malaria in neonatal period is considered as a differential diagnosis of numerous other infections. It is only mentioned when an infectious work-up has formally ruled out any other aetiology of sepsis in a neonate [19, 20]. However, given the resurgence of observational studies reporting the presence of asexual forms of *Plasmodium* in neonates of mothers infected with malaria on one hand, and of studies reporting malaria as the sole aetiology of neonatal infection in symptomatic infants on the other hand, congenital and neonatal malaria has received renewed interest from healthcare workers in recent years [20–25]. Therefore, to curb the burden of malaria globally with specific attention in endemic areas, it is important to accurately analyse epidemiological data with the aim of informing policy makers, and hence produce tailored public health interventions. The present systematic review with meta-analysis aims to provide a comprehensive overview of clinical malaria occurrence in neonates in endemic settings.

## Methods

This review was registered in the Prospective International Register of Systematic Reviews (PROSPERO with number CRD42020150124) and is reported according to the MOOSE (Meta-analysis Of Observational Studies in Epidemiology) guidelines [26].

### Search strategy and selection criteria

The following databases were searched up to October 30th, 2019: PubMed, EMBASE, Global Index Medicus, and Web of Science (Web of Science Core Collection, Current Contents Connect, KCI-Korean Journal Database, SciELO Citation Index, Russian Science Citation Index) to identify observational studies reporting on neonatal and congenial malaria with at least 30 participants. No restriction related to language was applied. The search strategy was designed for EMBASE and was modified to suit other databases (Additional file 1: Table 1). It was based on a combination of medical subject heading terms and text words related to congenital and neonatal malaria. The references of the retrieved articles were also screened with the aim to identify other potential data sources. Letters, commentaries, reviews, and editorials, as well as studies from which it was not possible to extract data on the prevalence of congenital and neonatal malaria were excluded.

### Malaria cases definition

Several definitions of neonatal and congenital malaria are used in the literature, but none has been consensually adopted [15, 27–29]. In this review we have considered neonatal malaria as: the presence of a positive parasitaemia with asexual forms of plasmodium associated with at least one symptom (fever, jaundice, anaemia, splenomegaly, vomiting, hepatomegaly, diarrhoea, restlessness, drowsiness, convulsions, poor feeding, cyanosis, pallor, respiratory distress) in a child less than or equal to 28 days [29, 30]. When it occurred in a neonate up to 7 days, it was considered as congenital malaria [27, 29]. Cases of incidental parasitemia, i.e. malaria-positive parasitaemia in asymptomatic neonates were not considered as neonatal or congenital malaria.

### Data extraction and management

Two investigators (CD and RNN) independently screened the titles and abstracts of the references found through the bibliographic search. Subsequently, the full texts of retained articles were uploaded and scrutinized for final inclusion. When the two investigators did not agree on the inclusion of an article, a discussion was held to reach a consensus.

With the help of a ready-made electronic data extraction sheet, two of the investigators (CD and RNN) independently extracted from the full texts, information in accordance with the objectives of the review. A third author then proceeded to cross-check and synthesize the extracted data. Data of interest included: surname of the first author, year of publication, country where the study was conducted, study design, sampling method, timing of data collection, proportion of males, specific characteristics of the study population, proportion of pregnant women who took intermittent preventive treatment for malaria, sample size and number of cases of clinical neonatal and/or congenital malaria.

To assess the methodological quality of each study, two investigators (CD and RNN) used an adapted version of the Joanna Briggs Institute tool for prevalence data [31].

### Data synthesis and analysis

The analyses were performed using the packages ‘*metafor’, ‘meta’*, and ‘*dmetar’* within the statistical software R (version 3.6.2). A DerSimonian and Laird random-effects model for meta-analysis was used to obtain the pooled prevalence. Prior to that, the variance of individual studies was stabilized with the Freeman-Tukey double arc-sine transformation [32]. Prevalence per 1,000 neonates (‰) were reported with their 95% confidence intervals. Cochran’s χ^2^ and I^2^ tests were used respectively to assess the presence and the amount of heterogeneity [33, 34]. I^2^ values of 25%, 50% and 75% were considered as representing low, medium and high heterogeneity respectively. Whenever substantial heterogeneity was found (I^2^ > 50%), a subgroup analysis was performed to determine its possible sources using the following grouping variables: regions (Africa and outside Africa) and malaria diagnostic method used (microscopy, antigen detection, molecular biology). Adjusted prevalence was computed using a multivariate meta-analysis to take into account the variance due to differences in diagnostic methods, and the variance due to region/country, in a hierarchical model. Funnel plot inspection and the Egger test (*p* < 0.10) were used as the two means of detection of publication bias [35]. A *p* value < 0.05 was considered as indicative of a statistically significant difference.

### Role of the funding source

This study received no funding. The corresponding author had full access to all the data in the study and had final responsibility for the decision to submit for publication. However, as a PhD candidate, the corresponding author is receiving a scholarship from UcLouvain.

## Results

### Study selection and characteristics

Following the bibliographical search, 1,961 studies were identified. After removal of duplicates, screening of titles and abstracts 245 studies were eligible and their full text assessed for final inclusion. Finally, 22 studies were retained contributing for 24 prevalence data (Additional file 1: Figure S1).

Nineteen (86.4%) studies were cross-sectional, while three (13.6%) were cross-sectional analysis of cohort studies. Studies were published between 1992 and 2015 and data collected between 1989 and 2013. Nineteen (86.4%) studies were conducted in Africa (all from sub-Saharan Africa), and the remaining three (13.6%) in Americas (Colombia, Peru), and South-East Asia (Indonesia). All countries outside Africa where in the control phase of malaria elimination, while those on the African continent were in endemic areas. Data collection and analysis was prospective in all studies except one. The most commonly used malaria diagnostic method was microscopy (95.4%, *n* = 21). The proportion of males varied between 46.2% and 63.7% as reported in eight studies. As reported in nine studies, the proportion of pregnant women who took intermittent preventive treatment for malaria (at least one dose) varied between 14.3% and 100%. Furthermore, the proportion of pregnant women with malaria during pregnancy varied from 9.6% to 57.1% as reported in four studies (Additional file 1: Table S2).

Most of studies used a representative sample size of the targeted population (81.8%), a probabilistic sampling (63.6%), and described the city where the study was conducted (95.4%). Precision (sample size < 385) was low in most studies (68.2%) (Additional files 1: Tables S3 and S4).

### Prevalence of congenital and neonatal malaria in Africa and outside Africa

**Fig. 1:**
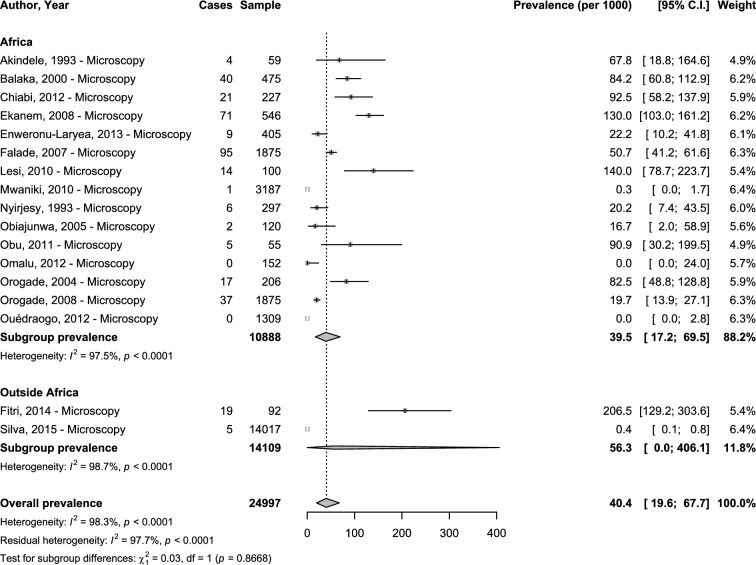
Meta-analysis prevalence of congenital malaria by region

Concerning clinical congenital malaria, only microscopy was used as diagnostic method. The overall prevalence was 40.4‰ (95%CI 19.6–67.7; 17 studies) with substantial heterogeneity (Fig. 1). The adjusted prevalence taking into account the variance due to difference in region/country (hierarchical model) was 33.7‰ (95%CI 6.9–77.2). The explained heterogeneity in this adjusted analysis was 0% for region and 98.9% for countries. There was no difference between the prevalence in Africa 39.5‰ (95%CI 17.2–59.5; 15 studies) and outside Africa 56.3‰ (95%CI 0.0–406.1), *p* = 0.867 (Fig. 1).

**Fig. 2:**
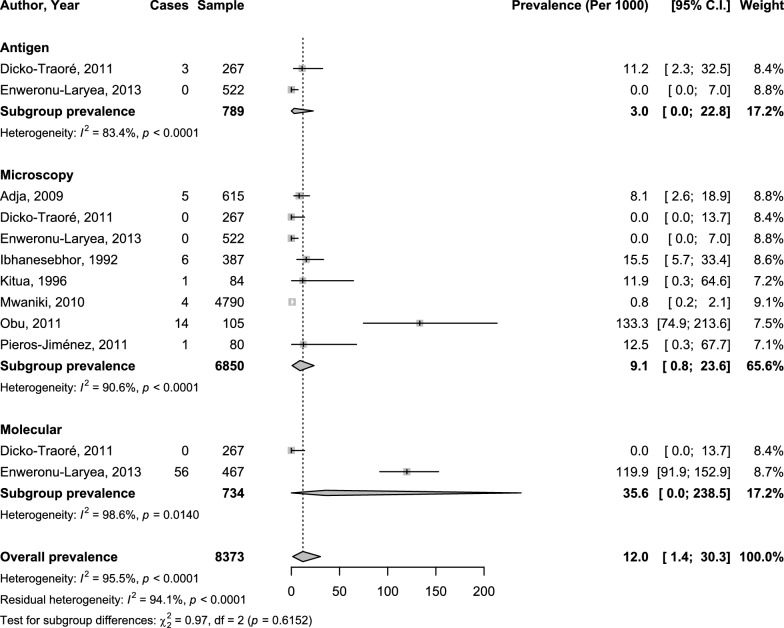
Meta-analysis prevalence of neonatal malaria by diagnostic technique

**Fig. 3:**
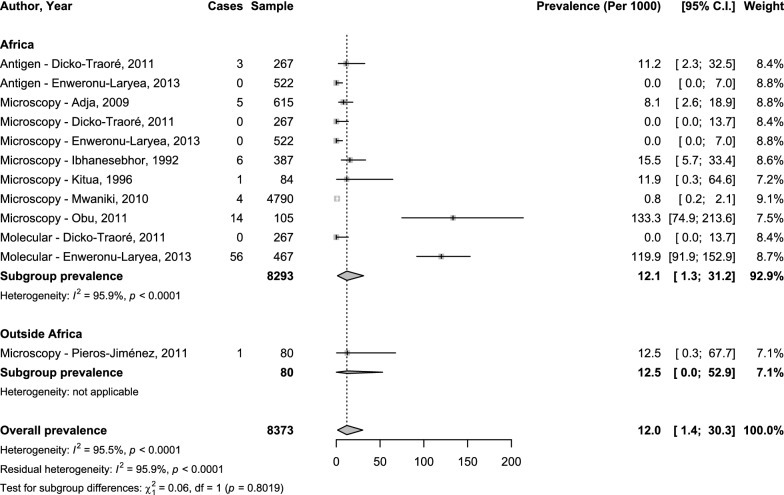
Meta-analysis prevalence of neonatal malaria by region

Concerning clinical neonatal malaria, the overall prevalence was 12.0‰ (95%CI 1.4–30.3; 12 studies) with substantial heterogeneity (Figs. 2 and 3). The adjusted prevalence taking into account the variance due to diagnostic methods and the variance due to difference in region/country (hierarchical model) was 12.9‰ (95%CI: 0.1-39.7). The explained heterogeneity in this adjusted analysis was 0% for region, 0% for diagnostic method, 89.3% for countries, and 5.8% for diagnostic method. The prevalence was 9.1‰ (95%CI 0.8–23.6; 8 studies), 3.0‰ (95%CI 0.0–22.8; 2 studies), and 35.6‰ (95%CI 0.0–238.5; 2 studies) with microscopy, antigen technique, and molecular technique respectively; with no significant difference (*p* = 0.615) (Fig. 2). There was no difference between the prevalence in Africa 12.1‰ (95%CI 1.3–31.2; 11 studies) and outside Africa 12.5‰ (95%CI 0.0–52.9), *p* = 0.802 (Fig. 3).

The funnel plot (Additional file 1: Fig. S2) for congenital malaria studies suggested asymmetry confirmed by the Egger test (*p* = 0.001). This was not the case for neonatal malaria where there was some symmetry in the funnel plot (Egger test *p *= 0.1004) (Additional file 1: Fig. S3).

## Discussion

This first systematic review with meta-analysis of 28,083 neonates living in 14 endemic malaria countries points out a high prevalence of clinical congenital and neonatal malaria, with a substantial heterogeneity not explained by the geographical location of the study endemic area and diagnostic method used. Considering 1,000 neonates, 40 may experience malaria clinical disease during the first 7 days of their life. After this period, for those aged less than 28 days, among 1000 neonates, 10 may experience malaria clinical disease.

As suggested by several observational studies, the prevalence of neonatal malaria appears to be lower than that observed in adults and children under 5 years of age. Indeed, according to WHO, 11.5% to 46.4% of the cases were thought to occur in the under 5 age group in sub-Saharan Africa [1]. The relatively low prevalence of clinical malaria in the neonatal period is probably multifactorial [36, 37]. The presence in the neonatal red blood cell of a high concentration of foetal haemoglobin that induces inadequate intraerythrocytic conditions for *Plasmodium* growth is considered one of the most plausible explanations [37–39]. The passively acquired neonatal immunity due to in utero transmission from the mother to the newborn could also play a role in its ability to not develop symptomatic forms of malaria even after exposure to *Plasmodium* [38]. As the kinetics of these passively acquired maternal antibodies show a progressive decline after three to six months, and that the amount of fetal haemoglobin decreases significantly at that age, children older than 6 months are more vulnerable to malaria [39–41]. This may explain why the prevalence of malaria increases in infants after the neonatal period [40, 41].

Although based on the analysis of two studies done outside Africa against sixteen done on this continent, findings of the current review suggest a lower prevalence of clinical congenital malaria in sub-Saharan Africa compared to regions outside the continent. Given the small number of studies conducted outside Africa, it is necessary to have more studies done on the occurrence of clinical congenital and neonatal malaria in endemic regions outside Africa in order to draw meaningful conclusions. Nevertheless, if these results are confirmed on a larger sample, they could be explained by the fact that in Africa, the prevalence of gestational and placental malaria is higher than elsewhere in the world, as reported by Desai and colleagues, who found that one out of four women in this region presents evidence of placental malaria infection during childbirth [42]. Similar results were also reported by the WHO in 2019, which estimated that 29% of pregnant women living in areas of high to moderate transmission in sub-Saharan Africa were exposed to malaria [1]. This triggers the synthesis and transfer of maternal antibodies against *Plasmodium* from mother to fetus during pregnancy and confers passive immunity against malaria to the neonates in this area, compared to other regions of the world where pregnant women are less exposed to malaria [42]. More importantly, a correlation has been shown between maternal and fetal antibody levels [36]. In addition, some studies have shown a higher prevalence of malaria parasitaemia in neonates in sub-Saharan Africa, compared to those living outside the region [24, 43–48]. They estimated the prevalence of congenital malaria, regardless of the presence of symptoms, to be between 10.8% and 54.2% in sub-Saharan Africa, 12.5% and 18.5% in the province with the highest burden in China [43, 47, 48]. In the USA, between 1960 and 2005, only 60 cases of congenital malaria were observed, and only a few number of cases were reported in Latin America [49–51]. The clinical picture of neonatal malaria is therefore influenced by the level of endemicity in the country. Children born from mothers living in high endemic areas, and hence passively received antibodies during pregnancy, are less likely to present clinical symptoms of malaria compared to those born from mothers living in non-endemic areas. In the latter, malaria may mimic a neonatal sepsis and represents a life-threatening condition.

Unsurprisingly, most of the studies included in this review are from sub-Saharan Africa, as in many previous reviews on gestational or congenital malaria [42, 43]. This is mainly due to the fact that 85% of the global burden of malaria is carried by nineteen sub-Saharan African countries and India, [1] possibly explaining their inclination to produce epidemiological data on clinical malaria in different age categories, including neonates.

In all the studies included in the current review, the diagnosis of malaria was made when the clinical picture was suggestive of infection and *Plasmodium* was the only infectious agent found in the biological exams. In low-income settings, given the presence of many other parasitic, bacterial and viral life-threatening infections, it is sometimes challenging to incriminate *Plasmodium* as the main aetiology of an infectious state when other germs are found in the blood especially in neonates. Therefore, to avoid pejorative outcomes, it may be necessary to adjunct anti-malarial therapy to medications meant to cater for other associated infectious agents.

In the current study, malaria prevalence data were pooled regardless of *Plasmodium* species. This is because only two studies reported the presence of non-*Plasmodium falciparum* malaria. The low number of studies reporting on non-*P. falciparum* malaria was probably due to the fact that most of our studies were from sub-Saharan Africa, where *P. falciparum* is the main aetiology of malaria and is more often associated with clinical manifestations, whereas in America and South-East Asia, *Plasmodium vivax* and *Plasmodium ovale* are more prevalent and less likely to produce severe clinical forms compared to *P. falciparum*.

The results of the current study support the need to consider malaria as a plausible differential diagnosis of fever in neonates, in areas where malaria is still endemic in general, and in sub-Saharan Africa in particular. They also call for the intensification of actions geared towards preventing neonatal malaria by reinforcing therapeutic and preventive measures for pregnant women, and lastly, for the elaboration of specific guidelines for the treatment of malaria in neonates.

Nevertheless, the results of this review must be interpreted in the context of some limitations. Firstly, not all of the included studies reported the proportion of women who experienced malaria during pregnancy and the proportion, who received intermittent preventive treatment. This made it difficult to assess the influence of malaria in pregnancy on the prevalence of clinical congenital and neonatal malaria. Secondly, only three of the studies included in the analysis were conducted outside sub-Saharan Africa, making it difficult to generalize the results to all malaria endemic areas. Thirdly, only two studies reported the presence of *P. vivax* in their sample, thus a subgroup analysis according to *Plasmodium* species was not conducted. Nevertheless, this study is the first to comprehensively synthesize epidemiological data on clinical congenital and neonatal malaria in endemic areas. Strong and robust methodological methods were used to minimize bias and produce the most accurate estimates. In addition, no restriction related to language or year of publication were applied, which allowed the analysis of all potentially eligible studies.

## Conclusion

Clinical malaria in neonatal period is not a rare event especially in endemic settings. There is a need to intensify efforts aimed at preventing neonatal malaria, by strengthening therapeutic and preventive measures for pregnant women, and to develop specific guidelines for the treatment of malaria during the neonatal period.

## Supplementary information

**Additional file 1: Figures and Tables**.

## Data Availability

All related materials are available in the appendix.
