# Supplementary material for: Epidemiology of clinical congenital and neonatal malaria in endemic settings: a systematic review and meta-analysis
Source: Malar J. 2020 Aug 28;19:312. doi: 10.1186/s12936-020-03373-8 (PMC7456021; doi:10.1186/s12936-020-03373-8)
Supplement: Supplementary file 1 — Additional file 1: Figures and Tables. [file 12936_2020_3373_MOESM1_ESM.docx]

# Epidemiology of congenital and neonatal malaria in endemic settings: a systematic review and meta-analysis

APPENDIX

**Supplementary Tables**

[Supplementary Table 1. Search strategy in EMBASE 2](#_Toc35581046)

[Supplementary Table 2. Characteristics of included studies 3](#_Toc35581047)

[Supplementary Table 3. Methodological quality of individual included studies 5](#_Toc35581048)

[Supplementary Table 4. Methodological quality of included studies (summary) 7](#_Toc35581049)

**Supplementary Figures**

[Supplementary Figure 1. Process of identification and selection of studies for inclusion in the review (PRISMA flow diagram) 8](file:////Users/drdanwang/Desktop/Review%20congenital%20malaria/DOssier%20mail%2022%20Mars%202020/CNM_Appendix.docx#_Toc35736226)

[Supplementary Figure 2. Funnel plot for prevalence data of congenital malaria 9](#_Toc35736227)

[Supplementary Figure 3.Funnel plot for prevalence data of neonatal malaria 9](#_Toc35736228)

Supplementary Table 1. Search strategy in EMBASE

|  |  |
| --- | --- |
| #1 | 'malaria'/exp OR malaria:ti,ab,kw OR plasmodium:ti,ab,kw OR paludism:ti,ab,kw OR 'marsh fever':ti,ab,kw |
| #2 | 'umbilical cord':ti,ab,kw OR congenital:ti,ab,kw OR neonat*:ti,ab,kw |
| #3 | #1 AND #2 |

Supplementary Table 2. Characteristics of included studies

| Author | Year | Design | Country | City(ies) | Year data collected | Setting | Timing of data collection | Age (in Years) | %Males | %IPT | %Pregnancy malaria | Fever | Diagnostic | Status of the country | Plasmodium s*p* |
| --- | --- | --- | --- | --- | --- | --- | --- | --- | --- | --- | --- | --- | --- | --- | --- |
| Adja (1) | 2009 | Cross sectional | Côte d'Ivoire | Yopougon | 2005-2006 | Hospital | Retrospective | NR |  |  |  |  | Microscopy | Endemic | Unclear |
| Akindele (2) | 1993 | Cross sectional | Nigeria | Ibadan | 1991 | Hospital | Prospective | NR |  | 21.4 |  | 100 | Microscopy | Endemic | falciparum |
| Balaka (3) | 2000 | Cohort | Togo | Lomé | NR | Primary health care setting | Prospective | NR |  |  |  |  | Microscopy | Endemic | falciparum |
| Chiabi (4) | 2012 | Cross sectional | Cameroon | Yaoundé | 2007 | Hospital | Prospective | 1 | 54.3 | 97 |  |  | Microscopy | Endemic | falciparum |
| Dicko-Traoré (5) | 2011 | Cross sectional | Mali | Bamako | 2006-2008 | Hospital | Prospective | 2.7 | 63.7 | 90.5 |  | 46.1 | Microscopy; Antigen detection; Molecular diagnosis | Endemic | falciparum |
| Ekanem (6) | 2008 | Cross sectional | Nigeria | Calabar | NR | Primary health care setting | Prospective | 1 |  | 88.7 |  | 37 | Microscopy | Endemic | falciparum |
| Enweronu-Laryea (7) | 2013 | Cross sectional | Ghana | Accra | 2008-2010 | Primary health care setting | Prospective | NR |  |  |  |  | Antigen detection; Molecular diagnosis | Endemic | falciparum |
| Falade (8) | 2007 | Cross sectional | Nigeria | Ibadan,Ilorin,Enugu, Kaduna, | 2003-2004 | Primary health care setting | Prospective | NR |  | 84.4 | 9.6 | 1.71 | Microscopy | Endemic | falciparum |
| Fitri (9) | 2014 | Cross sectional | Indonesia | Maumere | 2012-2013 | Hospital | Prospective | NR | 46.2 |  |  |  | Microscopy | Control-phase | Unclear |
| Herbert (10) | 2011 | Cross sectional | The Gambia | Banjul | 1998-1999 | Hospital | Prospective | NR | 54.3 | 14.3 | 57.1 |  | Microscopy | Endemic | falciparum |
| Ibhanesebhor (11) | 1992 | Cross sectional | Nigeria | Benin city | 1990 | Hospital | Prospective | NR | 50 | 100 |  | 100 | Microscopy | Endemic | falciparum |
| Kitua (12) | 1996 | Cohort | Tanzania | Idete | 1993-1994 | Primary health care setting | Prospective | NR | 48 |  |  | 1 | Microscopy | Endemic | falciparum |
| Lesi (13) | 2010 | Cross sectional | Nigeria | Lagos | 2002 | Primary health care setting | Prospective | NR |  |  |  |  | Microscopy | Endemic | falciparum |
| Mwaniki (14) | 2010 | Cross sectional | Kenya | Kilifi | 2002-2009 | Primary health care setting | Prospective | NR |  |  |  | 39.00 | Microscopy | Endemic | falciparum |
| Nyirjesy (15) | 1993 | Cross sectional | Zaire | Eastern Zaire | 1989-1990 | Hospital | Prospective | 2 |  | 70.00 | 49.50 | 6.00 | Microscopy | Endemic | falciparum |
| Obiajunwa (16) | 2005 | Cross sectional | Nigeria | Ile-Ife | 1997 | Hospital | Prospective | NR |  |  |  | 3.6 | Microscopy | Endemic | falciparum |
| Omalu (17) | 2012 | Cross sectional | Nigeria | Minna | 2010-2011 | Primary health care setting | Prospective | 1 | NR | NR | NR | NR | Microscopy | Endemic | falciparum |
| Orogade (18) | 2004 | Cross sectional | Nigeria | Kaduna | 2001-2002 | Primary health care | Prospective | 3.5 | 53 |  |  | 82.3 | Microscopy | Endemic | falciparum |
| Orogade (19) | 2008 | Cross sectional | Nigeria | Multicenter (unclear) | 2003-2004 | Primary health care | Prospective | 1 |  |  |  | 38.9 | Microscopy | Endemic | falciparum |
| Ouédraogo (20) | 2012 | Cohort | Burkina Faso | Koupela health district, Health District of Bousse | NR | Primary health care | Prospective | NR |  | 100 |  |  | Microscopy | Endemic | falciparum |
| Pĩeros-Jiménez (21) | 2011 | Cross sectional | Colombia | Urabá | 2005-2007 | Primary health care | Prospective | NR |  |  |  |  | Microscopy | Control-phase | vivax=80%  falciparum=20% |
| Silva (22) | 2015 | Cross sectional | Peru | Iquitos | 2011-2013 | Hospital | Prospective | NR | 52.00 |  | 40.00 |  | Microscopy | Control-phase | vivax=60% |

NR: Not reported, IPT: Intermittent Preventive Treatment,

NB: The value of IPT represented the number of women receiving at least one dose of IPT during pregnancy.

**List of included articles**

1. Akaffou E, Dick F, N’guessan R. Epidemiological study of the malaria at the neonatal period in the teaching hospital of Yopougon--Republic of Cote d’Ivoire. Mali Méd. 2009 Jan 1;24:36–9.

2. Akindele JA, Sowunmi A, Abohweyere AEJ. Congenital malaria in a hyperendemic area: a preliminary study. Ann Trop Paediatr. 1993 Jan 1;13(3):273–6.

3. Balaka B, Agbere AD, Bonkoungou P, Kessie K, Assimadi K, Agbo K. Paludisme congénital-maladie à Plasmodium falciparum chez le nouveau-né à risque infectieux. Arch Pédiatrie. 2000 Mar 1;7(3):243–8.

4. Chiabi A, Lendem I, Kobela M, Mah E, Tietche F, Tchokoteu P-F. Incidence de paludisme congénital dans deux services de néonatalogie à Yaoundé, Cameroun. J Pédiatrie Puériculture. 2012 Dec 1;25(6):301–8.

5. Dicko-Traoré F, Sylla M, Djimdé AA, Diakité AA, Diawara M, Togo B, et al. Le paludisme congénital et néonatal en Afrique subsaharienne, un évènement rare ? J Pédiatrie Puériculture. 2011 Apr 1;24(2):57–61.

6. Ekanem AD, Anah MU, Udo JJ. The prevalence of congenital malaria among neonates with suspected sepsis in Calabar, Nigeria. Trop Doct. 2008 Apr;38(2):73–6.

7. Enweronu-Laryea CC, Adjei GO, Mensah B, Duah N, Quashie NB. Prevalence of congenital malaria in high-risk Ghanaian newborns: a cross-sectional study. Malar J. 2013 Jan 11;12:17.

8. Falade C, Mokuolu O, Okafor H, Orogade A, Falade A, Adedoyin O, et al. Epidemiology of congenital malaria in Nigeria: a multi-centre study. Trop Med Int Health TM IH. 2007 Nov;12(11):1279–87.

9. Fitri LE, Jahja NE, Huwae IR, Nara MB, Berens-Riha N. Congenital Malaria in Newborns Selected for Low Birth-Weight, Anemia, and Other Possible Symptoms in Maumere, Indonesia. Korean J Parasitol. 2014 Dec;52(6):639–44.

10. Obu H, Ibe B. Neonatal malaria in the gambia. Ann Med Health Sci Res. 2011 Jan;1(1):45–54.

11. Ibhanesebhor SE. Clinical characteristics of neonatal malaria. J Trop Pediatr. 1995;41(6):330–3.

12. Kitua AY, Smith T, Alonso PL, Masanja H, Urassa H, Menendez C, et al. Plasmodium falciparum malaria in the first year of life in an area of intense and perennial transmission. Trop Med Int Health. 1996;1(4):475–84.

13. Lesi FEA, Mukhtar MY, Iroha EU, Egri-Okwaji MTC. Clinical presentation of congenital malaria at the Lagos University Teaching Hospital. Niger J Clin Pract. 2010 Jun;13(2):134–8.

14. Mwaniki MK, Talbert AW, Mturi FN, Berkley JA, Kager P, Marsh K, et al. Congenital and neonatal malaria in a rural Kenyan district hospital: An eight-year analysis. Malar J. 2010 Nov 6;9:313.

15. Nyirjesy P, Kavasya T, Axelrod P, Fischer PR. Malaria during pregnancy: neonatal morbidity and mortality and the efficacy of chloroquine chemoprophylaxis. Clin Infect Dis Off Publ Infect Dis Soc Am. 1993 Jan;16(1):127–32.

16. Obiajunwa PO, Owa JA, Adeodu OO. Prevalence of congenital malaria in Ile-ife, Nigeria. J Trop Pediatr. 2005 Aug;51(4):219–22.

17. Omalu ICJ, Mgbemena C, Mgbemena A, Ayanwale V, Olayemi IK, Lateef A, et al. Prevalence of Congenital Malaria in Minna, North Central Nigeria. J Trop Med [Internet]. 2012 [cited 2020 Jun 30];2012. Available from: https://www.ncbi.nlm.nih.gov/pmc/articles/PMC3157756/

18. Orogade AA. Neonatal malaria in a mesoendemic malaria area of Northern Nigeria. Ann Afr Med [Internet]. 2004 [cited 2020 Jun 30];3(4). Available from: https://www.ajol.info/index.php/aam/article/view/8332

19. Orogade AA, Falade CO, Okafor HU, Mokuolu OA, Mamman AI, Ogbonu TA, et al. Clinical and laboratory features of congenital malaria in Nigeria. J Pediatr Infect Dis. 2008 Jan 1;3(3):181–7.

20. Ouédraogo A, Tiono AB, Diarra A, Bougouma ECC, Nébié I, Konaté AT, et al. Transplacental Transmission of Plasmodium falciparum in a Highly Malaria Endemic Area of Burkina Faso. J Trop Med [Internet]. 2012 [cited 2020 Jun 30];2012. Available from: https://www.ncbi.nlm.nih.gov/pmc/articles/PMC3235890/

21. Piñeros-Jiménez JG, Álvarez G, Tobón A, Arboleda M, Carrero S, Blair S. Congenital malaria in Urabá, Colombia. Malar J. 2011 Aug 16;10:239.

22. Silva H, Laulate B, Coral C. [Congenital malaria in a hospital in Iquitos, Peru]. Rev Peru Med Exp Salud Publica. 2015 Jun;32(2):259–64.

Supplementary Table 3. Methodological quality of individual included studies

| Author | Year | Was the sample representative of the target population? (No sub-selection of the sample) | Sampling | Precision | Response rate | City was given | Numerator and denominator reported |
| --- | --- | --- | --- | --- | --- | --- | --- |
| Adja | 2009 | Yes | Systematic sampling | Acceptable | NR | Yes | Yes |
| Akindele | 1993 | Yes | Systematic sampling | Not acceptable (Sample size < 385) | NR | Yes | Yes |
| Balaka | 2000 | Yes | Systematic sampling | Acceptable | NR | Yes | Yes |
| Chiabi | 2012 | Yes | Systematic sampling | Not acceptable (Sample size < 385) | NR | Yes | Yes |
| Dicko-Traoré | 2011 | Yes | Systematic sampling | Not acceptable (Sample size < 385) | NR | Yes | Yes |
| Ekanem | 2008 | Yes | Systematic sampling | Not acceptable (Sample size < 385) | NR | Yes | Yes |
| Enweronu-Laryea | 2013 | Yes | Consecutive sampling | Acceptable | NR | Yes | Yes |
| Falade | 2007 | Yes | Systematic sampling | Acceptable | NR | Yes | Yes |
| Fitri | 2014 | Yes | Consecutive sampling | Not acceptable (Sample size < 385) | NR | Yes | Yes |
| Herbert | 2011 | Yes | Systematic sampling | Not acceptable (Sample size < 385) | NR | Yes | Yes |
| Ibhanesebhor | 1992 | Yes | Systematic sampling | Acceptable | NR | Yes | Yes |
| Kitua | 1996 | Yes | Systematic sampling | Not acceptable (Sample size < 385) | NR | Yes | Yes |
| Lesi | 2010 | Yes | Consecutive sampling | Not acceptable (Sample size < 385) | NR | Yes | Yes |
| Mwaniki | 2010 | Unclear | Consecutive sampling | Acceptable | NR | Yes | Yes |
| Nyirjesy | 1993 | Unclear | Consecutive sampling | Not acceptable (Sample size < 385) | NR | Unclear | Yes |
| Obiajunwa | 2005 | Unclear | Consecutive sampling | Not acceptable (Sample size < 385) | NR | Yes | Yes |
| Omalu | 2012 | Yes | Systematic sampling | Not acceptable (Sample size < 385) | NR | Yes | Yes |
| Orogade | 2004 | Yes | Consecutive sampling | Not acceptable (Sample size < 385) | NR | Yes | Yes |
| Orogade | 2008 | Yes | Systematic sampling | Not acceptable (Sample size < 385) | NR | Unclear | Yes |
| Ouédraogo | 2012 | Yes | Random sampling | Acceptable | NR | Unclear | Yes |
| Pĩeros-Jiménez | 2011 | Yes | Systematic sampling | Not acceptable (Sample size < 385) | NR | Yes | Yes |
| Silva | 2015 | No | Consecutive sampling | Not acceptable (Sample size < 385) | NR | Yes | Yes |

NR: Not reported

Supplementary Table 4. Methodological quality of included studies (summary)

| **Items** | **Assessment** | **n** | **%** |
| --- | --- | --- | --- |
| Was the sample representative of the target population? | No | 1 | 4.5% |
|  | Unclear | 3 | 13.6% |
|  | Yes | 18 | 81.8% |
| Sampling | Consecutive | 8 | 36.4% |
|  | Random | 1 | 4.5% |
|  | Systematic | 13 | 59.1% |
| Precision | Acceptable | 7 | 31.8% |
|  | Low (Sample size < 385) | 15 | 68.2% |
| Response rate | NR | 22 | 100 % |
| City was given | Unclear | 3 | 13.6% |
|  | Yes | 19 | 86.4% |
| Numerator and denominator reported | Yes | 38 | 100.0% |

Supplementary Figure 1. Process of identification and selection of studies for inclusion in the review (PRISMA flow diagram)

## Screening

## Eligibility

## Identification

## Included

Records screened on the basis of title and/or abstract
(n = 1,727)

Records excluded (clearly irrelevant)
(n = 1,482)

Records identified through database and manual searching
(n = 1,961)

Duplicates removed

(n = 234)

Studies included in qualitative synthesis
(n = 22 studies)

Studies included in quantitative synthesis (meta-analysis)
(n = 22 prevalence data)

Full-text articles assessed for eligibility
(n = 245)

Full-text articles excluded, with reasons (n = 223):

- 83 No prevalence data on CNM as defined in the review
- 39 Wrong design
- 4 Duplicates
- 14 No data extractable
- 9 Not neonatal population
- 55 Full text not available
- 4 Low sample size (< 30 participants)
- 15 impossible to extract data only for neonates

Supplementary Figure 2. Funnel plot for prevalence data of congenital malaria

Supplementary Figure 3.Funnel plot for prevalence data of neonatal malaria
